# The Significant Role of the Microfilament System in Tumors

**DOI:** 10.3389/fonc.2021.620390

**Published:** 2021-03-17

**Authors:** Xin Jiang, Yiming Qin, Liu Kun, Yanhong Zhou

**Affiliations:** ^1^NHC Key Laboratory of Carcinogenesis, Hunan Cancer Hospital and The Affiliated Cancer Hospital of Xiangya School of Medicine, Central South University, Changsha, China; ^2^Cancer Research Institute, Basic School of Medicine, Central South University, Changsha, China; ^3^Department of Neurosurgery, Brain Hospital of Hunan Province, Clinical Medical School, Hunan University of Chinese Medicine, Changsha, China

**Keywords:** microfilament system, actin skeleton, cancer, Arp2/3, anticancer drugs

## Abstract

Actin is the structural protein of microfilaments, and it usually exists in two forms: monomer and polymer. Among them, monomer actin is a spherical molecule composed of a polypeptide chain, also known as spherical actin. The function of actin polymers is to produce actin filaments, so it is also called fibroactin. The actin cytoskeleton is considered to be an important subcellular filament system. It interacts with numerous relevant proteins and regulatory cells, regulating basic functions, from cell division and muscle contraction to cell movement and ensuring tissue integrity. The dynamic reorganization of the actin cytoskeleton has immense influence on the progression and metastasis of cancer as well. This paper explores the significance of the microfilament network, the dynamic changes of its structure and function in the presence of a tumor, the formation process around the actin system, and the relevant proteins that may be target molecules for anticancer drugs so as to provide support and reference for interlinked cancer treatment research in the future.

## Introduction

Actin polymerization is essential for cell migration and various cellular biological processes. It is indispensable for actin to go through a nucleating process to evolve actin filaments before starting polymerization. Nucleating agents include the formin family and actin-associated protein 2/3 (Arp2/3) complexes. Arp2/3 complexes containing Arp1b and Arpc5l subunits are efficiently superior to Arpc1a and Arpc5l complexes in accelerating actin assembly (1). The nucleating factors include WASP, Scar/WAVE, WASH and WHAMM (2, 3). The actin system performs a key function in the process of cell migration (4). As the intracellular integration of cadherin and integrin adhesion and due to the collective migration of cells, the actin system continuously promotes the epithelial mesenchymal transition (EMT) of tumor cells through continuous rearrangement of actin, upregulation or downregulation of relevant proteins, and ubiquitination, even transferring. In the tumor-associated environment, the microfilament connection between cancer and immune cells also restrains the killing capability of immune cells, which puts the cancer cells in a state of tumoral immune escape. Therefore, both microfilaments and their associated proteins could be applied as potential therapeutic targets and lay a foundation for clinical application in approaching years.

In basic research, a fluorescence polarization microscope can show the position and direction of fluorescence molecules and can be used to analyze the conformation of actin skeleton proteins. However, the difficulty of real-time actin imaging is that actin has two forms, monomer and polymer, and it is in dynamic equilibrium. Therefore, the traditional GFP/RFP fluorescent group labeling often produces a diffuse signal. The newly developed selective F-actin probes Lifeact and Utr-CH can improve this problem. Lifeact can bind to both monomers and polymers but maintains the high affinity of F-actin, and Utr-CH only binds to F-actin. Many recent studies have further edited the Lifeact and Utr-CH probes to better locate F-actin (5, 6).

## Basic Function of the Microfilament Network and Associated Proteins

### Basic Function of the Microfilament Network

The actin cytoskeleton is an important subcellular filament system. Microfilaments, microtubules, and intermediate filaments make up the vast majority of the cytoskeleton. Actin is the most abundant protein in almost all cells. It is available to polymerize from globular subunits into microfilaments rapidly, which helps to regulate the movement of whole cell and intracellular transportation. In addition, microfilaments could generate the inner core of microvilli and produce division rings during cell division, providing support for molecular motors involved in cargo transportation and sliding microfilament contraction (7–9).

Integrin-mediated mechanotransduction is also associated with actin networks. To interpret the biological information in the extracellular matrix (ECM), cells transmit the traction force produced by myosin to the ECM through adhesion. Integrin is a transmembrane protein, which connects the ECM and the actin skeleton in the cell and transmits the rearward driving force of the aggregated actin network at the membrane bulge to the ECM through mechanical sensitive proteins, such as talin and vinculin. This kind of mechanotransduction affects downstream molecules, such as Rho GEFs, RhoA, Rho-related kinase (ROCK), etc. A high degree of ECM fossilization can affect the cascade reaction of FAK, Srk, phosphoinositide 3-kinase, and the JNK pathway, thus affecting the activation of the YAP/TAZ factor in the Hippo pathway, promoting cell proliferation and differentiation, and inducing the expression of prosurvival genes (10–12).

**Figure 1:**
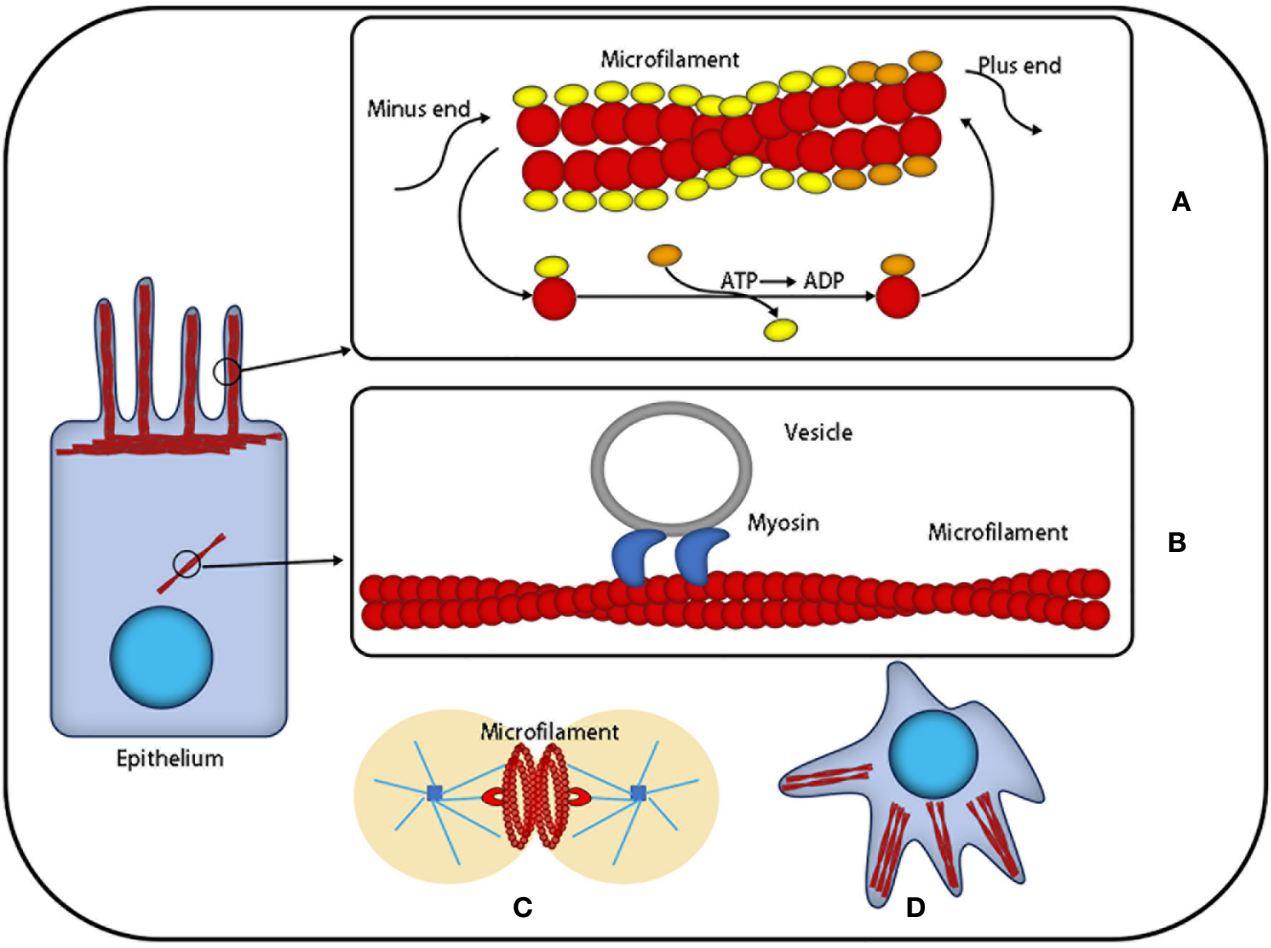
The function of microfilaments: Actin is the most abundant protein in most cells. It possesses the ability to rapidly polymerize from globular subunits into microfilaments, which regulates overall cell movement and intracellular transport. **(A)** Microfilaments can be used as a skeleton to support cells that are in the process of nonstop assembly and depolymerization. **(B)** Microfilaments can provide a platform for intracellular transport vesicles. **(C)** Microfilaments produce division rings during cell division. **(D)** Microfilaments can provide support for the morphological changes of cells.

Cell migration is essential for the development and various physiological processes in multicellular organisms. It is driven by specific processes and contractile actin filament structures. However, the types and relative contributions of these actin filament arrays vary with cell types and the cell environment. Besides individual migration behavior, collective cell migration is more effective, which indicates that cell-to-cell interaction occurs during collective migration (4, 13–15) (**Figure 1**). In addition, the destruction of the microfilament cytoskeleton is one of the symbols of cell morphological changes, inducing apoptosis *via* the PKB/survivin approach (15). Nuclear actin is involved in many DNA-related processes, including chromatin remodeling, transcription, replication, and DNA repair. It regulates the activity of RNA polymerase, the chromatin remodeling complex, and histone deacetylase (16, 17).

### The Function of Inter-relevant Microfilament Proteins

**Figure 2:**
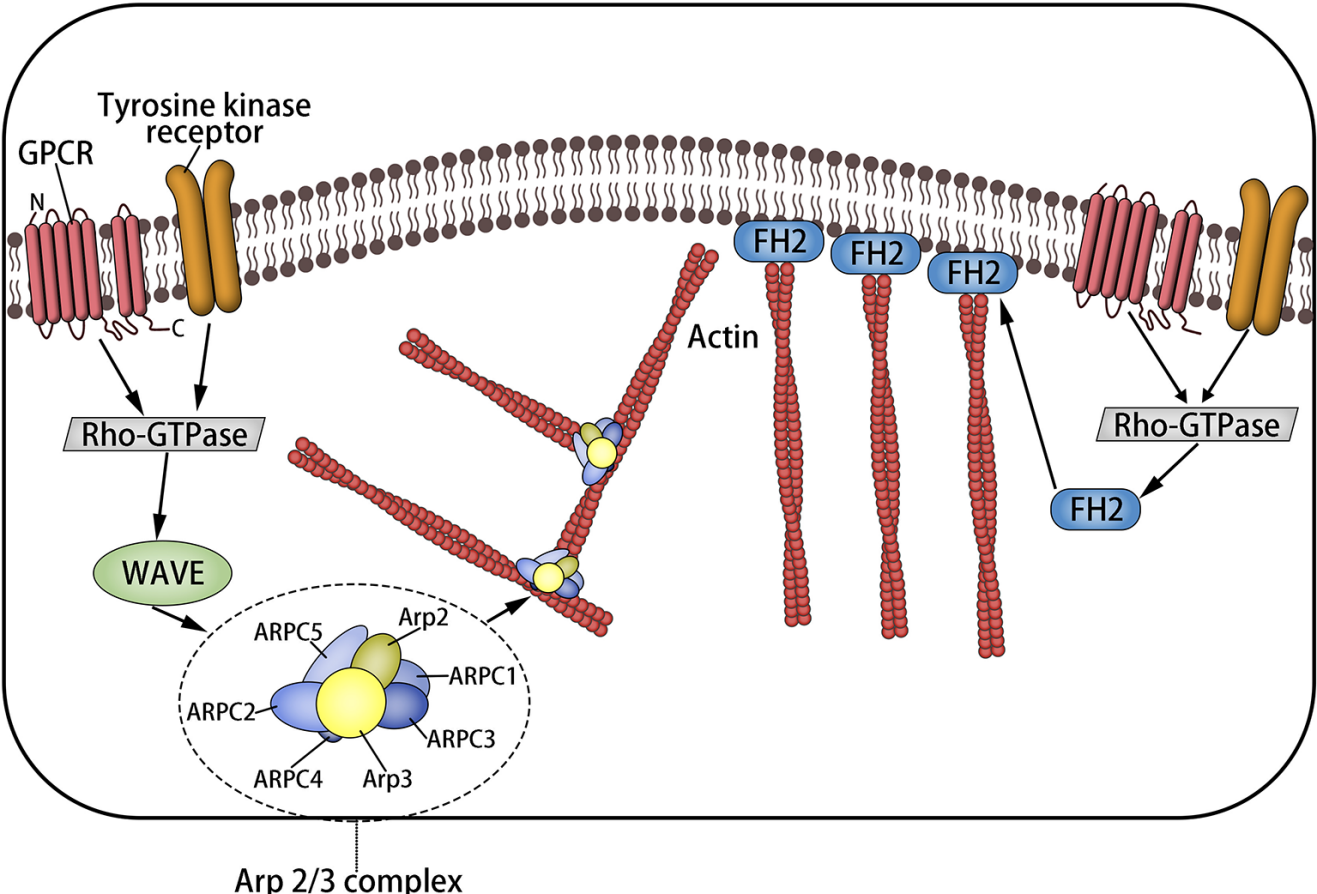
The formation of microfilaments: Rho-GTPases are a family of molecular switches that control the signal transduction pathway of eukaryotic cells. Rho promotes actin to aggregate into tension linear fibers and attach to the cell membrane through direct interaction with the target formin homologous domain mDia protein. It can also trigger the assembly of microfilaments by activating ROCK. Rac releases wave protein to activate Arp2/3, which elongates from the side of preexisting microfilaments to form the actin network.

Magnanimous inter-relevant proteins and regulatory elements interact with the actin skeleton, which makes it play a corresponding role. Rho GTPases are a family of molecular switches that control the signal conduction pathway in eukaryotic cells, including Rho, RAS, Cdc42, etc. (18). Rho promotes actin polymerization into linear fibers through straight interaction with the target formin homologous domain mDia protein (9, 19), in which formin protein is in a position to be activated separately but is self-inhibited (2) and is capable of touching microfilament components through activating ROCK. Rac releases wave protein to activate Arp2/3, which elongates from the side of preexisting microfilaments to create the actin network (1, 2, 19, 20) (**Figure 2**).

DOCK is a member of the GEF family (19). DOCK8 encodes a guanine nucleotide exchange factor that is highly expressed in lymphocytes and regulates the actin cytoskeleton. A scarcity of DOCK damages the migration, function, and survival of immune cells and affects the innate and adaptive immune response (21). Syndecans, a small family of heparan sulfate proteoglycans (HSPGs), which are a kind of transmembrane glycoprotein, regulate the microfilament system by interacting with Rho and Rac and mediate the regulation of calcium metabolism on microfilaments forthrightly (22). Taken as a cofactor, Actin interacting protein 1 (AIP1) preferentially promotes the decomposition and recombination of the actin filament modified by actin depolymerization factor (ADF)/cofilin (23, 24). Ezrin radixin moesin (ERM) protein is a sort of highly homologous protein that is indispensable for structural stability and integrity, maintaining the cell cortex by coupling transmembrane protein to the actin skeleton, and the binding is contributed by protein phosphorylation (25). Microfilament-associated protein palladin is an ezrin-related protein that possess a positive effect on actin skeleton assembly of dendritic cells (26). Drebrin is a widely distributed actin-related protein that is stored in many cell culture lines and tissues of epithelial, endothelial, smooth muscle, and nerve origin. It is rich in actin filaments related to plaque connection, and drebrin E is also essential for the remodeling of the actin cytoskeleton and the formation of cellular processes (27–30).

## Rearrangement in the Actin Skeleton and Metastasis of Tumors

### Changes to the Actin Skeleton in the Tumor Environment

**Figure 3:**
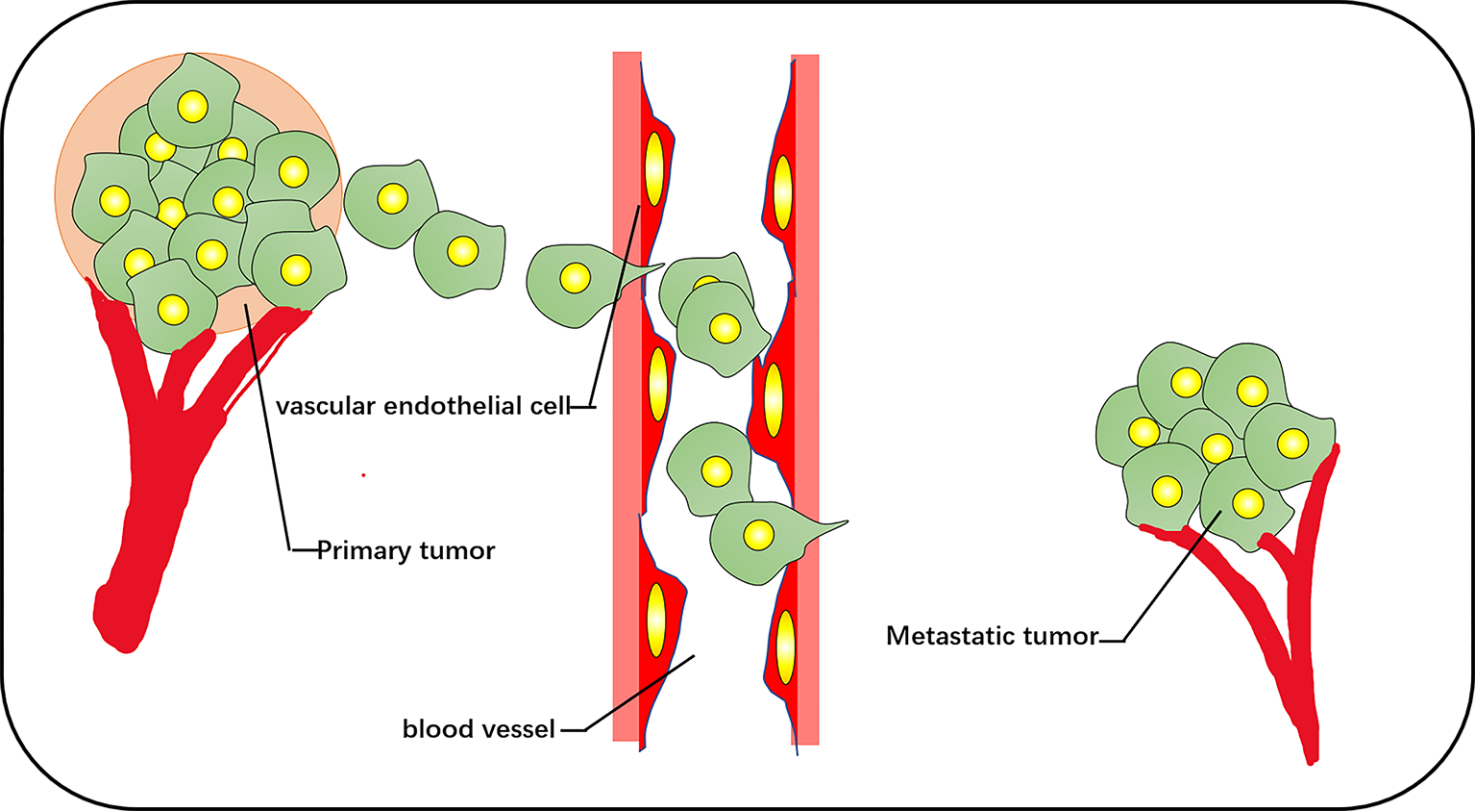
The vascular pathways of metastasis and diffusion of cancer cells include cell detachment from the primary tumor, invasion of blood vessels, and metastasis to other tissues for secondary tumor growth.

The increase of exercise activity, the increase of the cell proliferation rate, and the removal of cell–cell contact are the chief culprits of tumor origination, and metastasis is the cause of the largest-scale proportion of cancer deaths (31). Migration of cancer cells is composed of a series of discrete but continuous steps, which include detachment from the primary tumor, cell migration and invasion, intravenous injection, detachment by inducing the death of vascular cells, extravasation, and deterioration of the secondary tumor (32) (**Figure 3**). The change in cell diffusion is a representative feature of metastasis, which stimulates tumor cell movement to local and distant metastasis *in vivo*. The key factor is the dynamic reorganization of the actin skeleton, and microfilaments are attached to a special position on the plasma membrane. These adhesion structures connect the ECM with the nucleus through integrin. The reorganization of the actin cytoskeleton is crucial for the trans-differentiation of epithelioid cells into motile mesenchymal-like cells. This process is called EMT, which enables cells to elongate and move in a directional manner dynamically; the migration phenotype increases consequently (33, 34). EMT is associated with loss of the intercellular adhesion molecule E-cadherin, the destruction of intercellular junctions, and the acquisition of migration characteristics (including reorganization of the actin cytoskeleton and the activation of RhoA GTPase) (35). Transforming growth factor beta and the RhoA-LIMK2-confilin-1 signaling pathway regulate the actin skeleton, mediating the programming of EMT. EMT is the starting link of metastasis. When EMT occurs, the adhesion between tumor cells is reduced, and the abilities of movement and invasion are enhanced, which is conducive to the tumor cells leaving the primary lesion and entering the peripheral vascular or lymphatic system. Under the influence of the tumor microenvironment, tumor cells that have undergone EMT transformation reverse to restore the epithelial phenotype and regain adhesion ability, which is conducive to the homing and proliferation of tumor cells and the formation of metastasis. This reverse process of EMT is called mesenchymal epithelial transition (MET) (36). It is widely considered that the aggregation state of actin and the tissue of actin filaments are related to cell transformation. The proportion of polymerized actin in malignant keratinocytes is considerably reduced, and the stress fibers in cancer cells are concentrated into plaques at multiple points (37).

**Figure 4:**
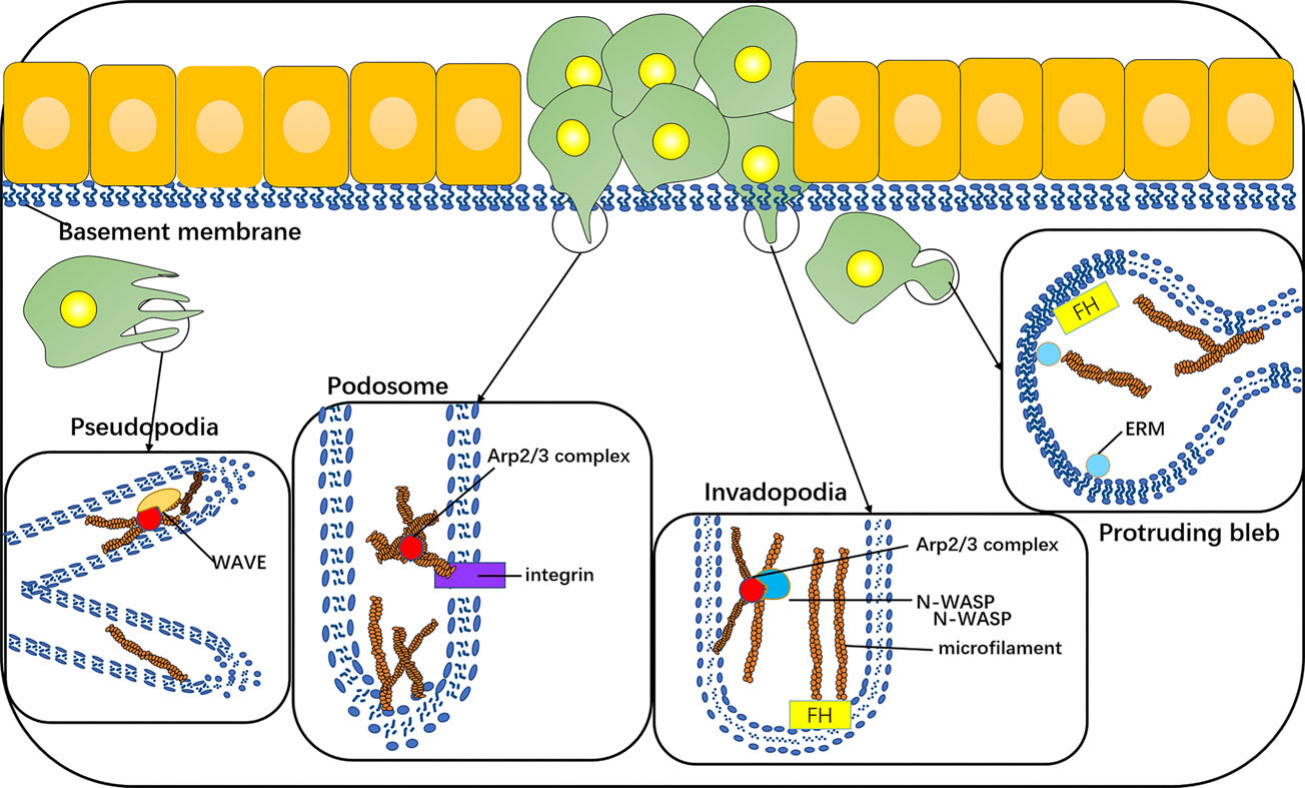
The invasive phenotype of cancer cells includes the formation of typical protuberant structures, such as protruding bleb, invadopodia, podosomes, or pseudopodia, which are dependent on the nucleation and assembly of actin filaments. Protruding bleb is a highly dynamic protuberance, which plays a role under the action of ERM and formin proteins. Invadopodia is a kind of actin-rich cell process, and its formation depends on the actin assembly driven by N-WASP-Arp2/3. The core tissue of a podosome contains an Arp2/3-dependent actin polymerization system, and its adhesion to the underlying matrix is mediated by integrins. Pseudopodia of cancer cells is lamellar, which depends on the polymerization and assembly of actin under the nucleation mechanism of WAVE-Arp2/3.

The invasive phenotype of cancer cells comprises the formation of typical protuberant structures, such as plasmalemma vesicles, invadopodia, podosomes, or pseudopodia, and all are dependent on the nucleation and assembly of actin filaments. The plasmalemma vesicle is an extremely dynamic protuberance due to the increase in hydrostatic pressure in the weak area of cortical actin, and the expansion of the plasma membrane, ERM, and formin proteins play a role (38–40), such as stem cell vesicle-like migration (39, 41). An invasive foot is a kind of actin-rich cell process, which is specially designed for the degradation of ECM. Its formation depends on the actin assembly driven by N-WASP-Arp2/3 (42, 43). A podosome is a special, dynamic, point-like structure enrichment in actin. There is an Arp2/3-dependent actin polymerization system inside its core tissue. Its adhesion to the underlying matrix is mediated by a ring containing integrins and integrin-related adhesion components (44). The pseudopodia of cancer cells is lamellar and dependent on the aggregation and assembly of actin under the nucleation mechanism of WASP-Arp2/3 (33) (**Figure 4**).

### The Association Between Cancer Cells and Other Cell Microfilaments in the Tumor Environment

Immune evasion is a characteristic of cancer, which causes cancer progress and invasion and shows resistance to chemotherapy. The adhesion of cytotoxic T cells (CTLs) to target cells is an important stage for effective cleavage of target cells, such as cancer cells. The binding and synaptic maturation of CTLs depend on the interaction of integrins on both sides of the synapses. Under normal circumstances, in CTLs and natural killer (NK) cells, the involvement of activated receptors induces phosphorylation of signal molecules at the proximal end of the membrane, forming a signal body containing many signal and adaptor molecules, which continuously stimulate actin polymerization and form a branched actin network around the synapse. The immune synapse (IS), also known as a supramolecular activation cluster (SMAC), is composed of concentric rings, which are divided into three parts: central SMAC (cSMAC), peripheral SMAC (pSMAC), and distal SMAC (dSMAC). The three parts form a bull’s-eye shape. In the immune synapse, CTL is located in the target cell base. Soon after the initial binding of the TCR–MHC-I complex, actin polymerization in CTL is activated, thus forming a circular branch chain actin network in dSMAC. This Arp2/3-mediated branch chain actin network is highly similar to the lamellar actin network of migrating cells, which allows CTL to diffuse on the surface of the target cell and supports the symmetrical retrograde movement of actin to cSMAC. pSMAC is composed of a sheet-like actin arc network, which mediates the radial symmetry of IS, and its assembly is mediated by formins. cSMAC is a low-density region of actin.

As mentioned, synaptic maturation depends on the interaction of integrins on both sides of the synapse. (1) The density of integrin increases with the increase of the density of LFA-1 at the boundary of the pSMAC and cSMAC. (2) Integrin-mediated adhesion rings around the cSMAC are also considered to be helpful in blocking the directional degranulation of the dissolved vesicles in the direction of the target cells. (3) The shear force produced by the reverse flow of actin supports the intercellular adhesion between LFA-1 and its ligand by inducing conformational changes of LFA-1 with high affinity. (4) Finally, LFA-1 and ICAM-1 can also be used as costimulatory molecules for CTL activation.

**Figure 5:**
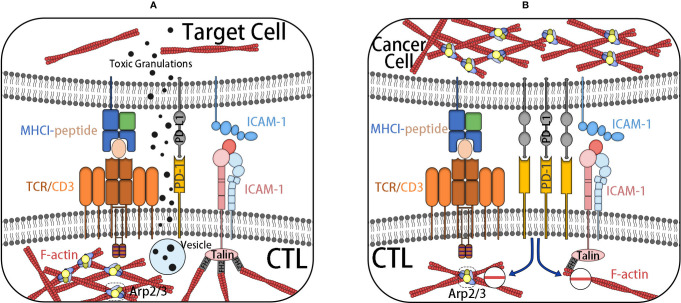
**(A)** IS are formed after CTL contacts with target cells. The initial binding of TCR–MHC-I complex activates actin polymerization in CTL. TCR–MHC-I complex is located in dSMAC, forming a network of branched chain actin, and a sheet actin network mediated by formin is formed at pSMAC where integrin is located. After the formation of an integrin-mediated adhesion ring at cSMAC, the targeted degranulation of dissolved vesicles in the direction of target cells is promoted. Finally, it induces the cell death of the target cell. **(B)** CTL, under the influence of inhibition signals, such as PD-L1, decreases the adhesion of the actin skeleton, inhibits the formation of actin-rich membrane processes, and reduces the transport rate of cytotoxic particles. It weakens the killing ability of CTL. At the same time, it increases the actin skeleton dynamics of cancer cells to prevent the formation of an adhesion ring and achieves the effect of immune escape.

In a nutshell, TCR–MHC-I interaction alone is not enough to activate CTL. After TCR activation, it is necessary to induce the change of CTL adhesion through the actin skeleton. Cancer cells can promote the actin skeleton changes of CTL, downregulate the RAS homologs Rho and Rac1, and promote the living star of Cdc42, resulting in negative regulation of integrin in CTL and subsequent mucosal and motor defects (45–47). At the same time, it can also increase the mobility of cancer cells to prevent the formation of a closed ring at the pSMAC/cSMAC boundary, thus reducing the transport rate of cytotoxic particles (**Figure 5**). Similarly, the actin cytoskeleton also plays an important role in the resistance of cancer cells to NK cells. NK cells need to form IS with cancer cells to inject cytotoxic mediators, such as perforin and granzyme B, into cancer cells to induce apoptosis. This key to formation requires significant accumulation of microfilaments (48).

In addition to the microfilament communication between cancer cells and other cells, it can also affect the configuration of the actin network through integrin-mediated mechanotransduction with ECM. Tumor progression is usually associated with pathological stiffness caused by extensive connective tissue proliferative responses. High density and hardness of ECM can promote integrin-dependent mechanotransduction and actin-mediated cell contraction, including subsequent YAP/TAZ activation, promoting cell proliferation, inhibiting cell apoptosis, leading to loss of cell contact inhibition, and promoting cell deterioration and transformation. This mechanical interaction between stroma and epithelial cells promotes tumor progression in a self-reinforcing feedback loop (10).

### The Promoting Effect of Microfilament-Relevant Proteins on Cancer

It is revealed that RhoA regulates several downstream targets, including ROCK, LIM kinase (LIMK) and cofilin. Dephosphorylation of cofilin enables actin depolymerization creation (49). Pre-mRNA processing factor 4B (PRP4) is overexpressed in HCT116 colon cancer cells by inhibiting RhoA activity and inducing cofilin dephosphorylation by inhibiting the Rho-ROCK-LIMK cofilin pathway and regulating the actin skeleton of cancer cells (50). However, the microfilament regulatory protein MENA accelerates RhoA activity and living cancer metastasis (51, 52). LMO2 is a crucial transcription regulator in the process of embryonic hematopoiesis and angiogenesis. The new effects of LMO2 are realized through its main cytoplasmic localization and interaction with cofilin1. High expression of LMO2 is positively correlated with lymph node metastasis in SCID mice, driving tumor cell migration and invasion and enlarging distant metastasis (53). Rho guanine nucleotide exchange factor 7 (ARHGEF7) is involved in cytoskeleton remodeling, which is very practical for cell motility and invasiveness, and it demonstrates frequent high-level gene amplification in colorectal adenocarcinoma metastasis (54). Syndecans is a small family of HSPGs, which interacts with Rho and Rac to regulate the microfilament system. Among them, SDC2 is a unique factor in cancer progression as it participates in the formation of EMT and relevantly increases the migration of a combination of tumor types, for instance, melanoma and nuclear fibrosarcoma, but it acts as a negative part in lung cancer and neuroendocrine tumor (55).

It is credible that the formation of an invasive foot depends on the actin assembly driven by N-WASP-Arp2/3 (56). In normal cells, N-WASP, WASH, WHAMM, and WAVE form branch actin networks on the dentate, endoplasmic, ER/Golgi surfaces and lamellar edge, respectively. Arp2/3 inhibitory proteins arpin, gadkin and PICK1 locally antagonize WAVE, WASH, and N-WASP in their respective positions. During the process of tumor progression and invasion, N-WASP is overexpressed and forms an invasion foot. Arp2/3 is mediated by the Plk4 Polo-box 1-Polo-box 2 domain, which phosphorylates Arp2/3 at the T237/T238 activation site (57).

Long nonencoding RNA (lncRNA) regulates ubiquitination of actin skeleton–related factors in cancer progression, such as ubiquitination of Ser3 and Tyr68 sites in cofilin, which slows down its activity (58) and promotes actin depolymerization. Small ubiquitin-related modifier (SUMO) family proteins are small ubiquitin-relevant proteins, which can bind to ubiquitin in a similar way as cell substrates on lysine residues. As for focal adhesion (FA), a large multiprotein complex (59, 60) combines ECM with transmembrane integrin molecules and connects with the actin cytoskeleton so that cells are able to produce traction force and play a central effect in cell migration, including FAK, talin, etc. Talin, a key component of FAs, can be post-translationally modified in MDA-MB-231 breast cancer cells and U2OS osteosarcoma cells through sumoylation by migration promoting activity (61, 62).

ERM is a class of highly homologous proteins. By coupling transmembrane proteins to the actin skeleton, ezrin regulation in breast cancer cells takes part in the interaction between adhesion molecules (CD44, ICAM, E-cadherin) and tyrosine kinase growth factor, epidermal growth factor (EGF), platelet-derived growth factor (PDGF), and their receptors (63). The microfilament-associated protein palladin is an ezrin-related protein that promotes the invasiveness of tumor metastatic cells by regulating the formation of invasive pods (64). ECM protein 1 interacts with mosesin (MSN), making it close to the cell membrane, promoting the translocation and phosphorylation of MSN membranes and promoting the formation of an invasive foot in breast cancer cells (65). With more miles to go, there are some microfilament-relevant proteins associated with cancer. Adenylate cyclase-associated protein (CAP) is a conserved actin regulatory protein. N-terminal mediates the Ras/cAMP signal. The C-terminal junction and separation of G-actin regulate the actin skeleton and assist in actin skeleton rearrangement. On the one hand, its upregulation supports metastasis; on the other hand, it contributes to inhibit the invasion of cancer cells through the FAK/ERK axis and Rap1 (66). Filamentous protein A (FLNA) is a well-known actin cross-linked protein, which has a dual role in cancer. When FLNA is localized in the cytoplasm, it functions to promote tumors by interacting with signal molecules. When it locates in the nucleus, it may interact with transcription factors to inhibit tumor growth and metastasis (67, 68). DMTN is a differentially transcriptional expressed gene; its downregulation regulates the actin cytoskeleton through Rac1 signal transduction and catalyzes the metastasis of colorectal cancer cells (69). The upregulation of plasminogen activator inhibitor 1 (PAI1) drives actin cytoskeleton rearrangement of triple negative breast cancer (TNBC) cells and increases migration (70). Cell adhesion molecules E-cadherin, phospholipase D (PLD), and various integrins regulate cell polarity, differentiation, proliferation, and migration through their close interactions with the actin cytoskeleton network (44, 71–73).

### Inhibition Effect of Microfilament-Associated Proteins on Cancer

Some microfilament-relevant proteins are not only a critical layer in promoting the progress of tumors, but they also stretch out in a complex, multifaceted way. For example, abnormal ubiquitination of an actin cytoskeleton regulatory factor may result in higher metastatic potential, but it is more likely to contribute to tumor inhibition. For example, E3 ligase hace1 is a tumor suppressor in NK cell malignancies and breast cancer (58). MicroRNA (miR)-185-5p inhibits F-actin polymerization by regulating advanced glycation end product–specific receptor (RAGE) and inhibits S100A8/A9-induced EMT of human breast cancer cells through the nuclear factor kappa B/snail signaling pathway (74). Although mammalian transparent-related formin2 (mDia2/Dia3/Drf3/Dia) assembles a dynamic F-actin cytoskeleton, which is the basis of tumor cell migration and invasion, studies disclose that cancer-associated fibroblasts (CAFs) increase breast cancer movement by inhibiting mDia2. Profilin I belongs to a small actin-binding protein family and is held responsible for helping extend actin filaments at the front of migrating cells (75). Traditionally speaking, profilin I is considered an important control element for actin polymerization and cell migration. However, profilin I was downregulated in breast cancer and other cancer cells. It may be due to the decrease of the actin filament flow fraction ratio and the polymerization slow-down rate by increasing the intracellular level of profilin I. Besides this, increasing the profilin level also leads to the decrease of single-cell speed and direction transformation (76).

There are some downregulated suppressor proteins as well, such as collapse response mediator protein-1 (CRMP1), a cytoplasmic phosphoprotein, which was primordially regarded to be the mediator of signal transduction protein 3A involved in axon differentiation during neurodevelopment. It plays a necessary part in EMT and metastasis inhibition in anterior adenocarcinoma cells by regulating actin polymerization, which discloses that CRMP targeted in actin tissue signaling may be a potential strategy for the treatment of prostate cancer metastasis (77). An actin-binding protein AIM1 is a key inhibitor of invasive phenotypes in primary and metastatic prostate cancer (78).

## Development of Anticancer Drugs for the Microfilament System

It is, thus, obvious that the rearrangement of the actin system has an effect on tumor progression and migration, which also induces microfilaments as an effective target for anticancer drugs. Subsequently, we are more likely to put forward some anticancer drugs for the microfilament system (79–81).

Microfilament-targeting drugs, such as cytochalasin and jasmine lactone, have been intensively and effectively exerted in clinical cancer treatment (82, 83), and staurosporin and curcumin have equiform effects to cytochalasin B, which may be on account of their ability to inhibit protein kinase C, a known microfilament assembly enhancer (84).

3-bromopyruvate (3-bp) is an alkylating agent and glycolysis inhibitor, which destroys the actin cytoskeleton and possesses the potential to affect the treatment of metastatic prostate cancer (85). Graphene inhibits the activity of the electron transfer chain, resulting in the decrease of ATP production and subsequent damage of F-actin cytoskeleton assembly, thus inhibiting tumor metastasis (86, 87). p53 can also regulate actin cytoskeleton remodeling in response to the extracellular microenvironment and play an anticancer role (88) other than inducing apoptosis of cancer cells. Linusorbs (LOS) can also delay the migration of C6 cells by inhibiting the formation of the actin skeleton (89).

Marine macrolide compounds, such as Sphinx lactone, swinholide a, hurghadolide a, and syctophycins, novel and effective anti-microfilament compounds, can bypass the multidrug resistance mediated by P-glycoprotein or MRP overexpression so as to cure drug-resistant tumors (90–93). Diethyl 2-(aniline methyl) malonate (dam) increased the basal activation of Smad2/3 and ERK and inhibited microfilament remodeling and the growth of cancer cells (94). Staurosporine (STS), a protein kinase inhibitor, is involved in cell death due to mitosis and discordant lamellar foot activity (95). Wihaferin A is mediated by annexin II to induce actin microfilament aggregation to treat cancer (96). Pentoxifylline (PTX; Hoechst) is a kind of microfilament depolymerization agent, which can significantly inhibit the lung homing of B16F10 cells. The microfilament network of cells treated with gyp was seriously collapsed, and the number of microvilli was reduced to a large extent (97).

Besides synthetic drugs, melatonin in the human body is the main secretion product of the pineal gland in the dark phase of the photoperiod and performs a cytoskeleton-regulating role in normal and cancer cells and would alter the microfilament phenotype of MCF-7 human breast cancer cells, from invasive transitional cells to dormant microfilament phenotypes into nontransitional cells (98).

## Existing Problems and Prospects

The microfilament system is composed of actin and its relevant proteins and elements. Actin-relevant proteins and factors have a variety of functions, such as transmitting activation or inhibition signals of the microfilament system; assembling actin; assisting actin polymerization; enhancing the stability of actin fiber; motivating its depolymerization by cutting actin fiber; and mediating the connection between actin and the cell membrane, ECM, or other cells. Actin does duty for the physiological processes of cell morphology, adhesion, movement, mitosis, differentiation, endocytosis, exocytosis, organelles, and the transportation of various substances in the cell. During the normal physiological function of actin and its relevant proteins, the abnormality of any component (upregulation or downregulation of expression, enhancement or inhibition of activity, abnormal structure or distribution, etc.) may lead to or quicken the occurrence of tumors. If these abnormal components can be rectified, the occurrence of tumor may be restrained.

At present, innumerable studies on microfilaments and cancer are being carried out all over the world. The principal research orientations are as follows: (1) Abnormal function of actin-relevant proteins, especially the RhoA-ROCK signaling pathway and Arp2/3 microfilament regulation system; (2) local adhesion of the microfilament system to cancer cells; and (3) the microfilament system and EMT process.

Alternatively, there is a noteworthy research direction: the vital influence of the microfilament cytoskeleton in the signal transduction between ECM and the nucleus. This significant research was carried out by Clubb et al. (99) but attracts little public attention currently. Nowadays, there are a variety of anticancer drugs for the microfilament system, such as cytochalasin, marine macrolides, staurosporin, and melatonin. However, most of them are still in the experimental stage; consequently, there is still a long way to go before clinical application. The microfilament system is an extremely complex and precise cell system, which takes precedence over any other systems in all stages of cancer occurrence. Its complexity bring about a lot of unknown mysteries in research. Therefore, further and more intensive research is required. However, it also provides a fundamental and alternative direction for the understanding of cancer and the research and development of anticancer drugs.

## Author Contributions

All authors listed have made a substantial, direct, and intellectual contribution to the work and approved it for publication.

## Funding

This paper was supported and conducted by the National Natural Sciences Foundation of China [grant number 81672685] and the Science and Technology Foundation Survey Project of Ministry of Science and Technology of China [grant numbers 2018FY100900 and 2018FY10090004].

## Conflict of Interest

The authors declare that the research was conducted in the absence of any commercial or financial relationships that could be construed as a potential conflict of interest.
